# A Systematic Review of Gender Disparity in the Authorship of Clinical Trials and Clinical Practice Guidelines in Various Medicine Subspecialties

**DOI:** 10.7759/cureus.54165

**Published:** 2024-02-14

**Authors:** Abhi C Lohana, Zubair Rahaman, Yaqub N Mohammed, Syeda D Samreen, Amit Gulati, FNU Shivani, Sakshi Khurana, Danesh Kumar, Sanjay Kirshan Kumar

**Affiliations:** 1 Internal Medicine, West Virginia University (WVU) / Camden Clark Medical Center, Parkersburg, USA; 2 Internal Medicine, University at Buffalo Jacobs School of Medicine and Biomedical Sciences, Buffalo, USA; 3 Internal Medicine, Western Michigan University Homer Stryker M.D. School of Medicine, Kalamazoo, USA; 4 Internal Medicine, Wayne State University, Detroit, USA; 5 Cardiology, Icahn School of Medicine at Mount Sinai, New York, USA; 6 Internal Medicine, Ascension Saint Joseph, Chicago, USA; 7 Radiology, NewYork-Presbyterian/Columbia University Irving Medical Center, New York, USA; 8 Internal Medicine, Henry Ford Jackson Hospital, Jackson, USA; 9 Gastroenterology, Sindh Institute of Urology and Transplantation, Karachi, PAK

**Keywords:** medicine, clinical, articles, authors, research, gender disparity

## Abstract

Authorship in clinical trials and clinical practice guidelines is considered prestigious and is associated with broader peer recognition. This systematic review investigated female representation among studies reporting authorship trends in clinical trials or clinical practice guidelines in different medicine subspecialties. Our search strategy yielded 836 articles, of which 30 met the inclusion criteria. Our findings indicate that females are severely underrepresented in authorship of clinical trials and clinical practice guidelines. Although the proportions of females may have improved in the past decade, the gains are marginal. Notably, studies in this domain predominantly focus on first/last authorship positions, and whether females are underrepresented in other positions as collaborative partners is currently unknown. Also, authorship trends in clinical trials or clinical practice guidelines of most medicine subspecialties besides cardiovascular medicine remain under-researched. Hence, standardizing the methodology for studying gender disparity in research output for comparative analysis between different subspecialties is as urgent as addressing the gender disparity in authorship.

## Introduction and background

Studies indicate that the pervasive gender disparity in academic research output [1-3] is not associated with the peer review process of medical/health journals [4-6]. However, women do not seem disadvantaged in peer review outcomes [4-6]. Thus, it may be hypothesized that the gender disparity in authorship may be driven by lower quality of female-led research, low number of female researchers, or both, all of which would indicate systemic problems in the medical workforce.

For instance, studies indicate that young female faculty members have more difficulty finding mentors than their male counterparts [7-10]. Mentorship opportunities have been identified as an important determinant of promoting and retaining females in academic medicine [10-11]. Thus, the lack of mentorship for early-career female researchers could lead to lower research funding [12-14], resulting in lower quality of research work and detrimentally impacting their representation in the authorship of research articles. Consistent with these observations, a meta-analytic study by Li et al. [15] showed that male physicians were 1.71 times more likely to hold federal research grants, 2.61 times more likely to hold leadership positions, published 17.20 more articles, had a higher h-index (by 5.97), and earned higher salaries (by $32,520) than female physicians [15]. Similarly, another study showed that women academic physicians across all specialties were less likely to hold an NIH grant or have a trial registered on ClinicalTrials.gov [16].

Ultimately, this would result in lower promotion odds and arrested career growth, as observed in several studies [16-19]. The "leaky pipeline" phenomenon in academic medicine has been widely reported [17-19], where fewer female researchers move up the academic hierarchy [15-16]. For example, although several Western countries have closed the gender gap in medical school enrolments for over a decade [20-22], males were 2.63 times more likely to be full professors in 2010-2020 than females, with similar trends within and outside North America [15]. Several factors have been attributed to the "leaky pipeline" from medical school to senior academic positions, such as higher teaching workload, work-life commitments, lower self-efficacy, perceived lack of leadership qualities, gender norms, discrimination, patriarchal organizational setup, sexual harassment, and self-undervaluing of skills, talents, or accomplishments [23-25]. In addition, some studies have observed an association between gender and the choice of specialization, which may reduce the proportion of women in certain specialties [26-27], affecting their representation in research output indicators.

Over the past decade, there has been tremendous interest in documenting the impact of these factors on authorship trends using bibliometric methodology [28]. For instance, studies demonstrate that females are severely underrepresented in the authorship of research articles [1-3], randomized controlled trials (RCTs) [29-30], clinical practice guidelines [31], clinical case reports [32], invited commentaries [33], and commissioned articles [34].

Among various study types, authorship in clinical trials and clinical practice guidelines is considered prestigious and is associated with broader peer recognition; therefore, the authorship trends in clinical trials and clinical practice guidelines may be particularly informative. For example, despite clinical trials being a collaborative effort, the first and last authors are often rewarded and promoted up the academic ranks [35-36]. Hence, the change in gender disparity in the authorship of clinical trials can indicate if the systemic problems, whatever they may be, that have led to the severe underrepresentation of females in authorship roles are being mitigated or exacerbated. On the other hand, typically, experts are invited to serve as panel members of clinical practice guidelines development [37], and changes in the gender disparity in clinical practice guidelines expert panels could indicate the level of disparity in opportunities to achieve peer recognition as subject matter experts.

Given that literature on authorship trends has been accumulating for over a decade, this study aimed to review reports of authorship trends in clinical trials or clinical practice guidelines systematically. Specifically, we were interested in the proportion of female authors reported in different medicine subspecialties, irrespective of the authorship position in clinical trials or clinical practice guidelines.

## Review

Materials and methods

This study was guided by the Preferred Reporting Items for Systematic Reviews and Meta-Analyses (PRISMA) guidelines [38].

Search Strategy

A systematic literature search from PubMed was conducted for relevant articles published from inception to November 1, 2023, for relevant articles using the following MeSH terms: (gender[tiab] OR female[tiab] OR women[tiab]) AND (authorship[tiab] OR bibliomert*[all]). The reference list of selected studies was also screened manually to identify additional eligible trials. The English synonyms were also used systematically as search syntax items in the databases to minimize the chances of missing relevant studies. In addition, the lists of bibliographies of the eligible articles and Google Scholar were manually searched to identify any potential reference for inclusion.

The article metadata comprising of PubMed ID (PMID), article title, author(s), journal of publication (including volume/issue/supplement details), publication date/year, and whenever available, the digital object identifier (DOI) were imported into a Microsoft Excel spreadsheet. Duplicate entries were checked using a similarity match based on PMID and DOI. No duplicate entries were found.

Study Selection and Data Extraction

Two authors (ACL and SK) independently screened studies in a two-phased approach, beginning with the primary screening of titles and abstracts. Our initial exploratory search revealed a wide range of bibliometric studies reported across various formats such as observational studies, case reports, reviews, news articles, letters, editorials, and comments. Consequently, we included all articles irrespective of their type while excluding retracted publications, errata, and non-English language articles.

The guiding criterion for the title and abstract screening was whether the article reported on gender disparity in authorship. Articles affirming this were coded for their respective sub-specialties, advancing to a thorough secondary screening. This stage involved a detailed review of the full texts, including any online supplementary data and appendices associated with the coded studies.

To resolve any discrepancies encountered during the screening stages, the investigators reached a consensus, occasionally consulting a third investigator (ZR) for further clarity.

Following this rigorous screening process, data extraction was meticulously carried out by two authors (AG and SK) who independently recorded details such as the author, period, sub-specialty, and inclusion criteria, in addition to the total number of articles (for clinical practice guidelines) and total number of authors (for clinical trials and clinical practice guidelines). A special focus was placed on quantifying the number/proportion of female authors in first, last, corresponding, or any authorship positions, ensuring a comprehensive analysis tailored to uncover the nuances of gender disparity in authorship within the medical research domain.

Inclusion and Exclusion Criteria

The eligibility of the articles obtained from the database and bibliography searches was assessed using the following inclusion criteria:

i. Studies involving gender disparity and clinical trials in medicine sub-specialties.

ii. Published primary studies inclusive of randomized control trials.

iii. Articles that examined the disparity in peer review, lack of mentorship, low citation, etc.

iv. The English language articles were prioritized for inclusion, and any other relevant articles could be translated into English.

Duplicated studies, commentaries, case reports, protocols, editorials, letters, conference abstracts, retracted, and articles in preprint were excluded. Articles not available online in full or from low-quality sources were also excluded.

Results

Search Results

The search strategy yielded 836 articles, of which 783 were eligible for title/abstract screening. Nearly a third of the studies (n=258; 32.9%) were excluded for not reporting gender disparity in authorship. Hence, 525 articles were retrieved for full-text screening, of which 12 (2.3%) were excluded for not reporting gender disparity in authorship, 328 (62.5%) were excluded for not including medicine subspecialties, and 156 (29.7%) were excluded as they did not specifically include or separately report data for clinical trials and clinical practice guidelines. Finally, 29 articles from the database search met our inclusion criteria. An additional article was identified through a reference list search of selected articles. Thus, 30 articles were included in this review.

Study Characteristics

Of the included studies, one reported authorship trends in both clinical trials and clinical practice guidelines [39], while more than 15 studies exclusively reported authorship trends in clinical trials [40-53] and 14 reported authorship trends in clinical practice guidelines [54-67].

The subspecialties of the studies that reported authorship trends in clinical trials were distributed as follows: cardiovascular medicine (n=4), gastroenterology (n=2), critical care (n=2), nephrology (n=1), neurovascular (n=1), rheumatology (n=1), sports medicine (n=1), exercise and rehabilitation (n=1), dermatology (n=1), and several medicine subspecialties (n=2). Overall, 12 out of 15 studies reported the proportion of females in first and last authorship positions, of which two also reported the proportion of female corresponding authors. Three studies only reported the proportion of female first authors. The detailed characteristics of studies reporting authorship trends in clinical trials are presented in Table 1.

**Table 1:** Characteristics of studies reporting authorship trends in clinical trials (CTs) that met our inclusion criteria. RCT = Randomized controlled trials

Author	Study period	Sub-specialty	Inclusion Criteria Used		Authors (Total)	Authorship position n (%)
			Design	Journal		
Reza et al. [39]	Jan 2001 to Dec 2016	Cardiovascular	CT	None (All heart failure RCTs indexed in PubMed or clinicaltrials.gov)	115	First: 11 (9.6) Last: 9 (7.8)
Denby et al. [40]	Jan 1, 2014, to Dec 31, 2018	Cardiovascular	CT	JAMA, Lancet, and New England Journal of Medicine	200	First: 18 (9) Last: 20 (10)
Whitelaw et al. [41]	Jan 1, 2000, to May 7, 2019	Cardiovascular	RCT	Impact factor ≥10	403	First: 63 (15.6) Last: 52 (12.9) Correspondence: 46 (11.4)
Mehran et al. [42]	Jan 1, 2011, to Oct 18, 2020	Cardiovascular	RCT	None (All PubMed-indexed RCTs)	6189	First: 1838 (29.7)
Foley et al. [43]	1971 to 2010	Gastroenterology	CT	Gastroenterology	223	First: 20 (9) Last: 19 (8.5) First/Last: 5 (2.2)
Bhatia et al. [44]	Jan 1, 1997, to Dec 31, 2017	Gastroenterology	CT	Gastroenterology, Gut, American Journal of Gastroenterology	3673 first and 3504 last authors	First: 804 (21.9) Last: 429 (12.2)
Romero et al. [45]	1981 to Dec 31, 2020	Critical Care	RCT	Only RCTs with significant differences in mortality among critically ill and perioperative patients.	340	First: 40 (11.8)
Chander et al. [46]	2000 to 2022	Critical Care	RCT	12 high-impact journals	1398	First: 344 (24.6) Last: 232 (16.6)
Shaik et al. [47]	Jan 1, 2000, to Apr 5, 2021	Neurovascular (stroke)	RCT/CT	None (All PubMed-indexed RCTs)	1944	First: 538 (27.7) Last: 289 (14.9)
Bagga et al. [48]	Jan 2015 to Dec 2019	Rheumatology	RCT	Impact Factors of > 3.0	603	First: 201 (33.3) Last: 159 (26.4)
Martinez-Rosales et al. [49]	Jan 1, 2000, to Sep 1, 2020	Sport Medicine	RCT	Q1 JCR journals in sports sciences published on behalf of a sports science scientific organization.	4811	First: 24.8% (9.7%-38.5%) Last: 16.8% (7.6%-22.3%)
Rinaldo et al. [50]	Apr 2017 to Mar 2022	Exercise & Rehabilitation	RCT	None (All PubMed-indexed RCTs)	5259	First: 2449 (46.6) Last: 1757 (33.4)
Ricardo et al. [51]	Jan 1, 2010, to Dec 1, 2020	Dermatology	RCT	None (All PubMed-indexed RCTs)	2401	First: 869 (36.2) Last: 646 (26.9%)
Shah et al. [52]	Apr 2012 to Mar 2017	Across 14 research domains	CT	Trials published by researchers funded or supported by the National Institute for Health and Care Research at Oxford Biomedical Research Centre	157	First: 65 (41.4) Last: 32 (20.4) Correspondence: 56 (35.7)
Rawlley et al. [53]	Jan 1, 2001, to Dec 31, 2016	All domains	RCT	First author from India	4056	First: 1198 (29.5)

Similarly, cardiovascular medicine (n=6) was the dominant subspecialty among studies reporting authorship trends in clinical practice guidelines, followed by gastroenterology (n=2), hepatology (n=2), and neurology (n=1), nephrology (n=1), rheumatology (n=1), pathology (n=1), endocrinology (n=1), and cross-discipline clinical practice guidelines (n=1). Seven of the 15 studies reported the proportion of female authors in clinical practice guidelines in any position. Five reported the proportion of females in the first and last authorship positions, of which two also reported the proportion of females in any authorship positions. Three studies only reported the proportion of female first authors. The detailed characteristics of studies reporting authorship trends in clinical practice guidelines are presented in Table 2.

**Table 2:** Characteristics of studies reporting authorship trends in clinical practice guidelines (CPGs) that met our inclusion criteria. AAN = American Academy of Neurology, AASLD = American Association for the Study of Liver Disease, ACC = American College of Cardiology, ACG = American College of Gastroenterology, AGA = American Gastroenterological Association, AHA = American Heart Association, APASL = Asian-Pacific Association for the Study of the Liver, BSG = British Society of Gastroenterology, CAP = College of American Pathologists, CCS = Canadian Cardiovascular Society, EASL = European Association for the Study of the Liver, ESC = European Society of Cardiology, JCS = Japanese Circulation Society, KASL = Korean Association for the Study of the Liver, and NGC = National Guideline Clearinghouse.

Author	Study Period	Subspecialty	Inclusion Criteria Used	Total Publications/Authors	Authorship position n (%)	
Reza et al. [39]	Jan 2001 to Dec 2016	Cardiovascular	2013 and 2017 ACC/AHA and 2016 ESC heart failure guidelines	273 CPGs 2031 Authors	First: 47 (17.2) Last: 34 (12.5)	
Tong et al. [54]	2008 to 2018	Cardiovascular	ACC guidelines	33 CPGs	Any: Median proportion of females was 22.2% (IQR 4.4–81.1)	
Rai et al. [55]	2006 to 2020	Cardiovascular	ACC/AHA, CCS, and ESC guidelines	203 CPGs 3433 Authors	Any: 811 (23.6)	
Rai et al. [56]	2001 to 2020	Cardiovascular	CCS guidelines	76 CPGs 1172 Authors	Any: 305 (26)	
Yamashita et al. [57]	2008 to 2022	Cardiovascular	JCS guidelines	101 CPGs 3461 Authors	Any: 192 (5.5)	
Dakhil et al. [58]	2002 to 2022	Cardiovascular	ESC guidelines	42 CPGs 862 Authors	Any: 174 (20.18)	
Bushyhead and Strate [59]	2007 to 2009	Gastroenterology	AASLD, ACG, and AGA guidelines	90 CPGs 460 Authors	Any: 97 (21.1)	
Li et al. [60]	2003 to 2022	Gastroenterology	Guidelines of global major gastroenterology societies	210 CPGs 461 Authors	First: 28/247 (11.3) Last: 21/214 (9.8)	
Ross et al. [61]	Jan 1, 2015, to Dec 31, 2020	Neurology	AAN-recommended guidelines	65 CPGs 707 Physician Authors	First: 12 (18)	
Adami et al. [62]	Jan 1, 2004, to Jan 1, 2019	Rheumatology	Global (Indexed in PubMed)	366 CPGs	First: 32.0% (95% CI 28.0–35.0%)	
Mantovani et al. [63]	Jan 1, 2014, to Dec 31, 2018	Hepatology	Global (Indexed in PubMed)	133 CPGs	First: 18.8% (95% CI 12.5–26.5%) Last: 14.6% (95% CI 9.0–22.9%)	
Tang et al. [64]	Jan 2008 to Sep 2022	Hepatology	AASLD, ACG, AGA, APASL, BSG, EASL, and KASL guidelines	103 CPGs 1096 Authors	First: 6 (33.3) Last: 2 (13.3) Any: 119 (43.3)	
Martin et al. [65]	2012 to 2021	Pathology	Current CAP guidelines	18 CPGs 275 Authors	First: 15 (14.6) Last: 21 (20.4) Any: 223 (20.3)	
Mantovani and Sartori [66]	Jan 1, 2016, to Dec 31, 2018	Endocrinology	Global (Indexed in PubMed)	90 CPGs	First: 28.9% (95% CI 19.8–39.4%)	
Merman et al. [67]	2012 to 2017	Cross-discipline	Published in the NGC (Jan 1, 2012, to Jul 10, 2016) and Guideline Central (Jul 11, 2016, to Dec 31, 2017)	545 CPGs 7134 Authors	Any: Median of 6 (3–8) women vs. 9 (5–13) men per guideline	
Chander et al. [68]	2000 to 2022	Nephrology	Impact Factor > 5	11 high-impact journals	1608	First: 328 (20.4) Last: 162 (10.0)

Data synthesis and discussion

Representation of Female Authors in Clinical Trials

In the cardiovascular medicine domain, the inclusion criteria of studies varied substantially to prevent a comparative analysis. For instance, Denby et al. [40] limited their analysis to just three general medicine journals. They reported 9% and 10% female representation in first and last authorship positions in clinical trials published between 2014 and 2018 [40]. Reza et al. [39] specifically analyzed gender presentation in heart failure trials published between 2001 and 2016 and reported 9.6% of females in the first and 7.8% in the last authorship positions. Whitelaw et al. [41] limited their analysis to clinical trials published in journals with an impact factor of over ten between 2000 and 2019 and reported 15.6%, 12.9%, and 11.4% female in first, last, and corresponding authorship positions. Finally, Mehran et al. [42] reported that 29.7% of the first authors were females in all cardiovascular-related clinical trials published between 2011 and 2020, indicating that female representation may have improved in the past decade. However, the study did not report gender representation in other authorship positions [42].

Similarly, although only two bibliometric studies reported gender disparity in the authorship of gastroenterology-related clinical trials, higher female representation was observed in the first and last authorship positions among clinical trials published between 1997 and 2017 (21.9% and 12.2%) [44] versus those published between 1971 and 2010 (9% and 8.5%) [43]. Both studies included clinical trials published in specialized journals. However, Foley et al. [43] limited their analysis to Gastroenterology, while Bhatia et al. [44] included clinical trials from Gastroenterology, Gut, American, and Journal of Gastroenterology.

Compared to nephrology, cardiovascular, and gastroenterology-related clinical trials, females were better represented in the first and last authorship positions of clinical trials related to other subspecialties. Among critical care, clinical trials published between 1981 and 2020, 11.8% of the first authors were females [45], while the proportion was 24.6% and 16.6% in the first and last authorship positions among clinical trials published between 2000 and 2022 [46]. Similarly, the proportions of females in the first and last authorship positions were 27.7% and 14.9% among neurovascular clinical trials (2000-2021) [47], 33.3% and 26.4% in rheumatology (2015-2019) [48], 28.4% and 16.8% in sports medicine (2000-2020) [49], 46.6% and 33.4% in exercise/rehabilitation (2014-2022) [50], and 36.2% and 26.9% in dermatology (2010-2020) [51] subdisciplines.

Two studies reported authorship trends combining clinical trials from various medicine subdisciplines: Shah et al. [52] reported that 41.4% of trials researchers funded or supported by the National Institute for Health and Care Research at Oxford Biomedical Research Centre had female first authors, while 20.4% had female last authors and 35.7% had a female corresponding author. Furthermore, in another study, Rawlley et al. [53] reported 29.5% of first-time female authors among RCTs in any medicine subspecialty. However, their inclusion criteria were limited to studies where the first author was affiliated with an institution in India.

These studies indicate that the representation of females in the authorship of clinical trials may have improved, especially in the past decade (2011-2020). Moreover, although the study by Shah et al. [52] was conducted in a specific context and may have limited generalizability, it may be highly indicative of the efforts of funding bodies to achieve better gender parity in authorship. Nonetheless, female authors remain severely unrepresented compared to male authors in all bibliometric analyses of clinical trials included in the current study.

Representation of Female Authors in Clinical Practice Guidelines​​​​​

Like clinical trials, female authors were severely unrepresented compared to male authors in all bibliometric analyses of clinical practice guidelines. Females comprised 22.2% (2008-2018), 20.18% (2002-2022), and 5.5% (2008-2022) of the authors (any position) of all clinical practice guidelines from the American College of Cardiology (ACC) [54], the European Society of Cardiology (ESC) [58], and the Japanese Circulation Society (JCS) [57], respectively. Rai et al. [55] assessed all clinical practice guidelines endorsed by ACC/AHA, CCS, and ESC between 2006-2020 and reported 23.6% female authorship in any position. In contrast, Reza et al. [39] specifically assessed female representation in the authorship of heart failure-related guidelines from ACC/AHA and CCS and reported 17.2% females in the first and 12.5% in the last authorship positions.

For the gastroenterology subspecialty, Bushyhead and Strate [59] reported 21.1% of females in any authorship position of clinical practice guidelines from discipline-related American societies, while Li et al. [60] reported 11.3% and 9.8% of females in first and last authorship positions in a combined analysis of guidelines of global major gastroenterology societies.

Two studies assessed authorship trends among hepatology-related clinical practice guidelines from global discipline-related scientific bodies but reported highly divergent results. Mantovani et al. [63] reported 11.3% and 9.8% of females in the first and last authorship positions among all PubMed-indexed clinical practice guidelines published between 2014 and 2018, respectively. In contrast, Tang et al. [64] reported 33% and 13.3% of females in the first and last authorship positions among clinical practice guidelines published between 2008 and 2022 by six hepatology-related scientific organizations (three American, one European, one British, and one South Korean).

In clinical practice guidelines related to neurology endorsed by the American Academy of Neurology between 2015 and 2020, females comprised about 18% of the first authors [61]. While even lower female representation in the first authorship position (14.6%) has been reported in pathology-related clinical practice guidelines published between 2012 and 2021, females were slightly better represented in the last authorship position (20.4%) [65]. Low female representation was also observed among nephrology-related RCTs published between 2000 and 2022, with 20.4% in the first and 10% in senior authorship positions [68]. However, a relatively better representation of females has been reported in the authorship of clinical practice guidelines related to rheumatology (32% among first authors during 2004-2019) [62] and endocrinology (28.9% among first authors during 2016-2018) [66].

However, one study assessed all clinical practice guidelines published in National Guideline Clearinghouse and Guideline Central between 2012 and 2017, irrespective of subspecialties, and reported a median of 6 (3-8) female authors versus 9 (5-13) male authors per guideline [67], highlighting the extent of the gender disparity in clinical practice guidelines authorship.

Summary of key findings

This systematic review has four significant findings. First, although the proportions of female authors may have improved in the past decade, the gains are marginal, and females remain severely unrepresented in the authorship of CT and clinical practice guidelines.

Second, the authorship trends in CT and clinical practice guidelines have been most well-researched in cardiovascular medicine. It is noteworthy that cardiology has an "outlier status" with the lowest proportion of females (15%) in the workforce compared to all other medical specialties [69-71]. Although the gender disparity in cardiology, in the case of both authorship of research output and workforce, is well-documented, the change has been marginal [72,73].

Third, the prevalent trend in the methodology of bibliometric analysis to study authorship trends in medicine heavily emphasizes the first and last authorship positions and, in the process, ignores the crucial collaborative contribution of authors who are not listed as first or last authors. The assessment of female representation in non-premier authorship positions is necessary to understand if females are also underrepresented as collaborative partners in medicine, as indications support this notion from bibliometric studies of academia in general [74,75]. While none of the studies reporting authorship trends in clinical trials in our sample reported trends in non-premier authorship positions, 60% (9/12) of the studies reporting authorship trends in clinical practice guidelines reported the proportion of females in any authorship position.

Ironically, the fourth important finding of this study was what was not found. Although our search strategies yielded studies reporting authorship trends in other medicine subspecialties such as emergency medicine (n=11), family medicine (n=7), immunology/rhinology (n=4), internal medicine (n=3), infectious disease (n=3), nephrology (n=2), pulmonary (n=2), and COVID-19 research (n=15), none of these studies specifically reported authorship trends in clinical trials and clinical practice guidelines. Hence, authorship trends in clinical trials or clinical practice guidelines of most medicine subspecialties are under-researched.

## Conclusions

Our systematic review emphasizes a concerning and persistent gender disparity in the authorship of clinical trials and clinical practice guidelines across various medicine subspecialties. While greater efforts are necessary to improve gender diversity in medical research, developing consensus on bibliometric analysis methodology for studying gender disparity in research output is necessary to allow comparative analysis of gender distribution between different subspecialties. Addressing these issues is crucial for fostering a more equitable and inclusive research environment in the field of medicine.
